# Factors influencing the behavior of bystanders to workplace bullying in healthcare—A qualitative descriptive interview study

**DOI:** 10.1002/nur.22228

**Published:** 2022-04-15

**Authors:** Sandra Jönsson, Tuija Muhonen

**Affiliations:** ^1^ Department of Urban Studies, Centre for Work Life and Evaluation Studies (CTA) Malmö University Malmö Sweden; ^2^ Department of School Development and Leadership, Centre for Work Life and Evaluation Studies (CTA) Malmö University Malmö Sweden

**Keywords:** bystanders, doctors, healthcare professions, nurses, qualitative research, workplace bullying

## Abstract

Workplace bullying is a severe problem that affects individuals, organizations, and society. Although there is a growing research interest in bystanders of workplace bullying, the rationale underlying bystanders' behavior in healthcare settings requires further investigation. The aim of the current study is to explore factors that influence the behavior of bystanders to workplace bullying in the healthcare sector. Qualitative semistructured interviews were conducted with 32 staff members in the healthcare sector in Sweden. Data were collected between March 2019 and September 2020 and were analyzed with thematic analysis. The participants experienced that bystanders of bullying, both colleagues and managers, were in many situations acting in a passive way. Organizational factors such as dysfunctional organizational culture and deficiencies in management affected how actively the bystanders could intervene. Additionally, a fear of negative consequences, lack of awareness of what was going on, bullying behavior being excused, and the bystander not being a member of the dominant group were social factors contributing to bystanders' passive behavior. For bystander intervention to be successful, the organization must consider bullying as a serious issue, take action, and show support for both the target and the bystander.

## 1 BACKGROUND

Workplace bullying is a severe problem that affects individuals, organizations, and society (Karatuna et al., 2020; Salin & Notelaers, 2020). Einarsen et al. (2011) define workplace bullying as harassing, offending, or socially excluding someone or negatively affecting their work. For the label bullying to be applied to a particular activity, interaction, or process, the bullying behaviors must occur repeatedly and regularly (e.g., weekly) and over a period of time (e.g., approximately 6 months). According to a recent systematic review of bullying among healthcare employees, up to one in four healthcare professionals were exposed to regular bullying (Lever et al., 2019). Workplace bullying in the healthcare sector has negative consequences for organizations and their patients, targets of bullying, and bystanders. As in the general population of workers, healthcare workers that have been bullied are more likely to report that they intend to leave their job in the near future (Evans, 2017). Nurses have also reported that workplace bullying negatively affects patient safety and contributes to medication and treatment errors (Oh et al., 2016; Wright & Khatri, 2015). Given the shortage of nurses and physicians and high turnover rates in the healthcare sector (Jönsson et al., 2021), it is important to elucidate the factors involved in bullying to create a safe and inclusive work environment.

Acts of bullying are often viewed as dyadic interactions between the victim and perpetrator, a perspective that overlooks the important roles of bystanders (Paull et al., 2012). The term bystander is used to describe not only an actor who is a passive witness or observer, but also an individual who has the potential to intervene in the bullying situation (Ng et al., 2020). Bystanders are by far the largest group exposed to bullying situations, with studies reporting that more than 80% of employees have witnessed workplace bullying (Pouwelse et al., 2021). This finding has prompted several researchers to highlight the importance of bystander action in tackling bullying (Pouwelse et al., 2021). Based on a typology developed by Paull et al. (2012), bystander behaviors can be categorized based on whether they are active or passive, and constructive or destructive, as follows: active constructive (e.g., defending the target), passive constructive (e.g., empathizing with the target), active destructive (e.g., collaborating with the perpetrator), or passive destructive (e.g., ignoring or avoiding the situation).

Previous studies have elaborated on the reasons why bystanders might choose not to intervene in workplace bullying situations or why their responses may vary over time. For instance, it can be difficult for bystanders to know *how to help* the victim (van Heugten, 2011; Keashly & Jagatic, 2003). Not knowing how to support or help the victim can make bystanders frustrated over their inability to intervene, resulting in anger toward the organization for not controlling the perpetrator (Keashly & Jagatic, 2003). In addition, bystanders may not think or feel that they have the *responsibility* to intervene (Mulder et al., 2016) or may consider the negative acts as *fair treatment* because of socialization into the workplace culture (Leymann, 1990; Ng et al., 2020). In other cases, bystanders may not recognize the *severity* of a bullying situation (Tracy et al., 2006) or they may only *see or observe certain isolated actions* (Einarsen et al., 2020). Studies focusing on the individual and more general aspects of bystander behavior have increased knowledge in this area. However, more attention needs to be paid to organizational settings to understand bystander behavior in a bullying situation.

*Organizational culture* has been identified as a critical factor for bystander action. In a study focusing on bystander intervention in healthcare, Thompson et al. (2020) concluded that the efficacy of bystander interventions is decreased in organizational cultures lacking understanding of bystander action, and where individuals reporting mistreatment are seen as “troublemakers.” Newcomers are typically socialized into the prevailing organizational culture, including how they are expected to think and act (Schein, 2010). Regarding organizational culture, healthcare workplaces have been reported to be characterized by organizational silence (Gaffney et al., 2012). Thus, even if employees have knowledge of certain issues and problems within the organization, they may not dare to speak up or inform their superiors (Morrison & Milliken, 2000). This indicates that employees may feel that voicing concerns would have no effect, or because they feel that doing so would have negative consequences on their work situation.

The roles of managers in relation to the awareness of bullying and bystander intervention have been studied to some extent. Previous studies reported that individuals who experienced bullying reported that their managers tolerated the bullying behavior (Einarsen, 1999; Farrell, 2001; Namie, 2008). Other researchers found that some organizations foster bullying behavior through inaction (Speedy, 2006). In one study focusing on bullying and ethical dilemmas among nurse managers, participants indicated that every time they were made aware of the behavior, they took action (Lindy & Schaefer, 2010). However, managers indicated that staff was not informed about when or what action was taken to address bullying behaviors. This may contribute to the perception that the bullying was ignored or tolerated (Lindy & Schaefer, 2010).

Although there is a growing research interest in bystanders of workplace bullying, there is a need for further investigation concerning bystander behavior and the rationale underlying the behavior (Thompson et al., 2020). The current study adds valuable insight to research on workplace bullying by analyzing bystander behavior in relation to the identified bullying situation and the organizational context of healthcare.

## 2 METHODS

### 2.1 Aim

The aim of the current study is to explore factors that can influence the behavior of bystanders to workplace bullying in the healthcare sector.

### 2.2 Design and sample

Regarding the exploratory aim of the study, a qualitative descriptive design was applied. The goal of qualitative descriptive design can be described as creating a comprehensive summary of events by staying close to the data and the surface of words and events (Sandelowski, 2000). Semistructured interviews were conducted with 32 staff members with different occupations in the healthcare sector (see Table 1). The majority of the participants were women, and the mean age was 49.29 years (standard deviation = 11.47; range: 28–70 years). The participants worked in different medical areas, including radiology, psychiatry, pediatrics, orthopedics, anesthesia, emergency care, palliative care, surgery, primary care, gynecology, and internal medicine. The purpose of including different occupations and clinical settings was to obtain a broader understanding of bystander behaviors in different healthcare contexts.

**Table 1:** Demographics of the participants

Characteristic	*N*	Characteristic	*N*
Gender		Occupation	
Women	24	Nurse	14
Men	8	Assistant nurse	2
		Doctor	12
Age (years)		Physiotherapist	3
Mean: 49.29		Technician	1
Range: 28–70			

Participants were recruited from a previous questionnaire study in which they had expressed interest in participating in an interview study focusing on bystanders to workplace bullying. The individuals that had shown interest in participating in the study were contacted by one of the researchers either by phone or email. Here, they were informed about the project and the aim of the study. They also received practical information about the upcoming interview. If they were interested in participating, interviews were booked. All participants were employed at a large organization responsible for all public healthcare in one region in Sweden.

### 2.3 Data collection

The interviews were conducted individually by the two authors between March 2019 and September 2020. The duration of the interviews ranged between 40 and 95 min and were conducted either face‐to‐face in the participants' workplace (N = 7), or via Zoom, Teams, or telephone (due to the Covid‐19 pandemic). The interviews were based on an interview guide including questions about the participants' background, their experiences of witnessing workplace bullying, and how they had acted in the bullying situations. All of the interviews were recorded with informed consent and were later transcribed verbatim. [Correction added on 25 April 2022, after first online publication: The second and third sentences of this paragraph have been corrected from “…and they were conducted in the participants' workplace via Zoom, Teams, or telephone. Face‐to‐face interviews were not possible because of the COVID‐19 pandemic.” to “…and were conducted either face‐to‐face in the participants' workplace (N = 7), or via Zoom, Teams, or telephone (due to the COVID‐19 pandemic).” in this version.]

### 2.4 Data analysis

Qualitative thematic analysis was applied when analyzing the data (Braun & Clarke, 2006). The authors individually read through the verbatim interview transcriptions several times to gain an overall impression of the entire material, and units of meaning related to the study aim were labeled with codes. Thereafter, the identified codes were discussed and revised, and a qualitative thematic analysis was conducted separately by coding the material according to the revised codes. In accordance with the research questions and analytic framework, we applied a model of inductive thematic saturation of the data with a focus on the identification of codes/themes (Saunders et al., 2018).

### 2.5 Rigor

The consistent use of the interview guide increased the dependability of the data. The analysis of the interviews was performed in close interaction between the authors. Discussions about the coding and analysis continued until consensus was obtained. During the analysis, the authors went back and forth to the original texts to verify that nothing had changed in meaning or was overlooked. Illustrative quotations were used when presenting data findings for credibility purposes (Lincoln & Guba, 1985).

### 2.6 Ethical considerations

Informed consent was obtained from all participants included in the study. All procedures were performed in accordance with the ethical standards of the Swedish Research Council (Good Research Practice, 2017).

## 3 RESULTS

### 3.1 Workplace bullying in the study context

The participants described bullying situations from both the target and bystander perspectives. The harassing behaviors included: silent treatment, social exclusion, or isolation of colleagues because of their way of working, slander, gossip, spreading rumors about a lack of competence or poor performance, being ignored, being made to feel invisible, being ridiculed, condescending comments, denial of assistance or help when needed, overly harsh criticism, or having competence questioned. Thus, the harassing behaviors were mainly psychological in nature, and mostly work‐related. More overt behaviors, such as yelling and personal attacks, were rarely described. Some situations were described as starting with more subtle negative behaviors that escalated over time into more explicit harassment that continued for a long period of time. A vast majority of participants had resigned and moved to another unit or hospital.

Participants described workplace bullying as being conducted horizontally, vertically (both upward and downward), and across professional groups. As examples of vertical upward bullying, doctors described situations where the perpetrator was a nurse. Another example was a situation in which a small group of assistant nurses was bullying the unit manager. In this case, the bullies undermined the manager on a regular basis and openly questioned her competence. Horizontal bullying was described by all professional groups and was specifically evident in situations in which an individual came to a new workplace when they had recently graduated or were relatively inexperienced.

### 3.2 Overarching themes

The final analysis of the data resulted in the following three overarching themes: 1. bystanders' behaviors; 2. organizational factors influencing bystander behavior; and 3. social factors influencing bystander behavior. Each theme consisted of subthemes, which are presented in Table 2.

**Table 2:** Themes and subthemes

Themes	Subthemes
Bystanders' behaviors	Active intervention from colleagues Social support from colleagues
Organizational factors influencing bystander behavior	Dysfunctional organizational culture Deficiencies in management
Social factors influencing bystander behavior	Fear of negative consequences (high costs) Failure to notice and/or to deal with the situation Bullying behavior is being excused Bystander not a member of the dominant group

### 3.3 Bystanders' behaviors

The participants' perceptions indicated that bystanders, both colleagues and managers, acted in a relatively passive way when they witnessed bullying situations. However, there were also some examples of bystanders intervening in an active and constructive way.

#### 3.3.1 Active intervention from colleagues

Two participants experienced some bystanders as relatively active. Situations were described in which bystanders had confronted the perpetrator or reported the situation to the manager to mitigate the effects of bullying or to make it stop.

*People felt that their perceptions and feelings were confirmed when I, as a witness, came forward and described what I saw and said that it was not acceptable behavior… I have also reported bullying to a manager when colleagues have been affected*. Nurse, IP24

This nurse identified that other colleagues had witnessed the bullying behavior but taken a passive stance. The nurse opted to engage in an active bystander response to create a different outcome.

#### 3.3.2 Social support from colleagues

Several participants reported receiving emotional social support from their colleagues, particularly if they were exposed to the same kind of behavior. In some cases, colleagues showed sympathy and support privately but were silent in staff meetings when the targets were expecting their support. The targets wished that their colleagues had been more active and confirmed the target's experience.

*And we all were sitting down and made observations (of the bullying behavior). When you come as a newcomer… nobody says anything, so I didn't say anything either… I was passive and I felt bad about it*. Physiotherapist, IP19

The above extract illustrates that it is even more difficult to speak up if no one else is intervening, causing individuals to become socialized into the organizational culture and a culture of silence.

### 3.4 Organizational factors influencing bystanders' behavior

Workplace bullying takes place in an organizational setting, and various aspects, including organizational culture and management, play an important role. Considering the organizational context is essential for understanding bystander behavior.

#### 3.4.1 Dysfunctional organizational culture

Some participants indicated that organizational silence was part of the culture in their organizations. Employees who highlighted issues in the work environment, or made suggestions about alternative ways of working, risked being punished.

*[Our] job is a calling – you don't want to complain, you just want to do your job, and do a good job. It is organizational silence: I mind my own business and you mind yours*. Doctor, IP25

In this kind of organizational culture, there was no discussion or tolerance of differences; rather, people were expected to conform to existing ways of working and thinking. This type of culture prevented both targets and witnesses from reporting harassing behaviors.

*There was some kind of weird friendliness. It was not authentic. Everybody was behaving correctly, but there was no warmth. It was not genuine… everybody was on their toes*. Nurse, IP23

Workplace bullying behavior was perceived as part of the organizational culture, which newcomers became socialized into. Those who were unable to adapt left the organization, while those who stayed believed that the same situation was occurring at all workplaces. In this way, the harassment became “normalized,” and the dysfunctional culture was reproduced.

*It is very strange, it is like people are schooled into this. It is a system sitting in the walls somehow, that is inherited further*. Doctor, IP27

*This kind of behavior is something one witnesses every day, so you become indifferent*. Nurse, IP1

This illustrates how the experience of witnessing negative behaviors over time changes the way employees think and react to them. Conflicts and tensions between different occupational groups can also arise because of the demarcation or blurring of professional boundaries, such as those between nurses and doctors, or those between assistant nurses, nurses, and physiotherapists.

*It is this lack of clarity – who is supposed to do what?… Maybe they (assistant nurses) find it convenient that somebody else does what they are supposed to do*. Physiotherapist, IP21

Unclarity or lack of understanding of different professional roles and the division of work can contribute to negative behaviors among employees. It can also influence the organizational culture and limit how individual bystanders to act.

#### 3.4.2 Deficiencies in management

Several bystanders had reported the harassment to their manager, but in many cases, this did not lead to any intervention or action.

*My colleague and I took up this with our manager to let them know that there was something sick/dysfunctional going on… The manager said: “What do you want me to do about it?” *Physiotherapist, IP19

The feeling of not being able to help the victim and not getting support from the manager was experienced as frustrating.

*In the meetings, we could see how the bullies were sighing over the target, while the manager didn't intervene at all*. Nurse, IP15

In this case, the manager was regarded as weak, and as not working actively with the staff and the work environment. Furthermore, participants reported that it was difficult to act when the bully was their manager. In some cases (e.g., a manager bullying a co‐worker), reporting resulted in the bystanders becoming targeted themselves. In some instances, the manager was a close friend of the bully, meaning that reporting the harassing behavior was not seen as worthwhile.

*… I feel angry toward our manager, who allowed this behavior to go on*… *The manager gave the impression that she was listening and understanding, but afterwards I realized that she didn't really care at all*. Nurse, IP13

Situations in which the manager initially appears to listen but then takes no action and demonstrates that they do not care tend to violate employees' trust and respect.

### 3.5 Social factors influencing bystanders' behavior

Participants reported many explanations regarding why bystanders choose to intervene or not. While some aspects were connected to the organizational context in a more general sense, others were related to the social context.

#### 3.5.1 Fear of negative consequences (high costs)

Bystanders perceived that it was dangerous to act because they were afraid of becoming targets themselves or that acting would escalate the current bullying situation. This fear was based on their own observations and experiences, witnessing situations that worsened when a manager or the victim brought up the issue in a staff meeting, or when the target confronted the bully directly.

*It often gets worse if you act to bring it out in the open. Then you have started something bigger… The situation does not get any better if you take it up with the manager, because then you are regarded as a whistle‐blower by the bullies*. Nurse, IP1

From an individual perspective, there was a fear of being socially excluded, or being offered less attractive work tasks or working hours. In situations where one's manager was the target, other issues emerged. As bystanders, participants reported that it was difficult to know how to deal with the situation. In the example below, the respondent was concerned about whether or not standing up for the manager would undermine her position.

*I feel like I can't undermine her in any way. She is not saying anything, and here I come, starting to say something. I don't want to diminish her… at a meeting I addressed the issue from a more general perspective*. Nurse IP18

#### 3.5.2 Failure to notice and/or to deal with the situation

Participants noted that bystanders might witness only minor instances of subtle harassing behavior and therefore fail to see that it was conducted systematically. Because it can take time to realize what is going on and the process may escalate, participants reported that it is difficult for an individual bystander to act. One participant reported that bystanders did not wish to exaggerate the situation or appear to be “reporting” on a colleague or manager unless the situation was experienced as severe.

*In the beginning you don't think that it is bullying, because that sounds quite serious to me… but when it starts building up, escalating and enduring, then you realize*. Nurse, IP7

As bystanders, participants reported that they often did not know how to deal with the situation. When the target was a manager, acting became even harder.

*I would say that this has been going on for six months, Maybe it started earlier, but I wasn't aware of it until six months ago. I think it has escalated and you are thinking, how should I deal with this situation?* Nurse IP18

*But what could I do? I was already exposed myself so… It was really a dead‐end situation. One did not know what to do. Either you endured or quit the job*. Nurse, IP15

Not knowing what to do create a feeling of frustration and helplessness. When voicing concerns did not help, the only two options left were to deal with the situation or to exit the organization.

#### 3.5.3 Bullying behavior is being excused

In some cases, the bystanders' reporting was futile because the bully's negative behavior was excused as they were highly competent people on whom the organization depended.

*It took a long time for me to figure out his behavior, because I saw him as a role model, a very competent nurse… He was the most competent nurse on the ward, in spite of the negative sides*. Nurse, IP7

Participants reported that there was a certain reluctance to identify individual colleagues as bullies. Furthermore, some bullies' behavior was excused because they had problems in their private lives.

*Her husband was sick, so people felt sorry for, even though she was not a nice person towards her colleagues… except those who were part of her clique*. Nurse, IP15

#### 3.5.4 Bystander not a member of the dominant group

Participants reported that the bully was often not acting alone but was a leading figure in a dominant group in the workplace. The group was often described as consisting of 3–4 members, where one played a more prominent role. In one case, the group had a close relationship with the manager.

*They were a team; we outsiders called them the Gang*… *And they were also friends with the manager, so who would listen to us*? Nurse, IP13

Participants reported that, as bystanders, it was difficult to intervene when they were in a less powerful position and when they could not rely on their colleagues' support.

*This group was like a cult, but they were a highly qualified group… It is incredibly difficult to act when those involved have very high competence*. Doctor, IP29

It was difficult for bystanders to act when they were dependent on the bullies' skills and competence in daily work situations.

## 4 DISCUSSION

The current study aimed to explore factors that influence the behavior of bystanders in response to workplace bullying in the healthcare sector, highlighting the complex dynamics among perpetrators, victims, bystanders, and the organizational context.

Regarding the described bullying behaviors, the experienced behaviors in this study were characterized as subtle, psychological, and work‐related. These behaviors are relatively common and not specific to this sector (Spector et al., 2013). In addition, these subtle negative behaviors tended, in some cases, to escalate over time into more intense harassment, leading the targets to resign and move to another workplace. Here, respondents' feelings of being “forced” to move to another workplace despite enjoying their current workplace and working with patients, were experienced as frustrating. This finding may be connected to the shortage of nurses and physicians and the high turnover rate, which has been issued in the sector for years (Jönsson et. al., 2021).

Because the healthcare sector is known for its hierarchical structure, it might be expected that workplace bullying would also follow a vertical pattern. However, the described bullying was performed horizontally (e.g., among nurses, especially targeting newcomers), vertically (e.g., managers could be both perpetrators and targets), and across different professional groups (e.g., assistant nurses targeting physiotherapists). Thus, there was no clear pattern regarding the direction or the involvement of different professions in the bullying situation. The horizontal nature of bullying has been seen in other studies as well indicating that perpetrators of bullying in nursing primarily consist of nurse colleagues or supervisors (Castronovo et al., 2016; Wilson, 2016). In addition, bullying from supervisors has also been highlighted and discussed in the bullying literature. Fink‐Samnick (2017) describes that healthcare organizations are made up of social hierarchies that set the stage for bullying situations. Bullying may be used as a strategy by some managers to pressure employees to get work accomplished faster, but in the long run will have a negative impact on morale and motivation (Ferris et al., 2007).

Turning the focus to the bystanders, even though there were a few reports of bystanders intervening in an active and constructive way (Ng et al., 2020; Paul et al., 2012), the participants mainly perceived bystanders as being passive either in a constructive way by privately showing social support and empathy for the target, or in a more destructive way by ignoring or avoiding the situation (Ng et al., 2020; Paull et al., 2012). This is in line with other studies focusing on bystanders in healthcare, showing a lack of direct action by witnesses of bullying (Thompson et al., 2020, discussion). This kind of passive behavior can be explained by the more general phenomenon of not knowing how to help the victim (van Heugten, 2011; Keashly & Jagatic, 2003), not feeling responsible for intervening (Mulder et al., 2016), considering the bullying as fair treatment (Leymann, 1990; Ng et al., 2020), or not seeing the severity of the situation (Tracy et al., 2006). However, it can also be related to organizational and social factors that are specific to the context of healthcare.

The one potentially important aspect is socialization into the different healthcare professions. Besides mastering professional skills (e.g., as a nurse or a physician, the socialization process involves the internalization of organizational and social behavior as part of the development of professional identity). In this study, the bullying behaviors appeared to be embedded in a dysfunctional organizational culture, in which newcomers were socialized into the prevailing culture (Schein, 2010). An example of this was the experience of a culture of silence (Gaffney et al., 2012; Morrison & Milliken, 2000), which made it hard for both targets and bystanders to act, and to find ways to deal with situations involving bullying.

This situation may also be related to healthcare management. The act of raising a concern with an employer about potential bullying can be seen as an important aspect related to strategies designed to manage workplace bullying (Thompson & Catley, 2018). In the current study, although some participants reported that they informed the manager about ongoing bullying, in most cases, nothing happened in terms of intervention (Lindy & Schaefer, 2010, discussion). At times, the perpetrators' behavior was excused because they were considered to be valuable staff members that the organization was dependent upon. Because of a lack of healthcare staff, the harassing behavior might be tolerated to avoid a staffing shortage.

Other social factors influencing bystander intervention included the fear of negative consequences (Báez‐León et al., 2016), failure to notice and/or deal with the situation (Keashly & Jagatic, 2003; van Heugten, 2011), bullying behavior being excused, and bystanders not being part of the dominant group (MacCurtain et al. (2018). Participants described situations in which both colleagues and managers knew that bullying and harassment existed, but nobody dared to speak up. Participants reported fear of negative consequences, such as being excluded both from the workplace and private social activities, or fear of becoming the next victim if they intervened.

Furthermore, informal power structures may make the situation even more complex, (Ashforth et al., 2000). These processes made it more difficult for bystanders to intervene and potentially change an ongoing destructive situation. In many cases, the bystanders did not believe that turning to their manager would change the situation because of loyalties and dependencies between management and the perpetrator(s). In addition, high turnover among managers enabled informal leaders (e.g., those who had been employed at the ward for a long period) to take over.

In most cases, participants reported that workplace bullying was not something that was discussed in the organization and that there appeared to be a lack of policy focusing on preventing or managing workplace bullying. In addition, participants felt that it was important for managers to be present and visible in the ward to prevent bullying, reporting that “harassment does not exist if the manager does not allow it,” intentionally or unintentionally.

Thus, the current results highlight the importance of clear policies and managers having appropriate knowledge and competence to contain and preferably prevent bullying in healthcare organizations.

### 4.1 Limitations

The participants in the current study represent different professions and units in the healthcare sector, increasing the transferability of the study results. However, the findings of the current qualitative interview study should be further investigated in a more comprehensive questionnaire study in the healthcare sector, preferably with a longitudinal design. This could enable causal analyses of the complex relationships between organizational and social factors in relation to bystander behavior in the healthcare sector.

## 5 CONCLUSION AND IMPLICATIONS

The results of the current study indicate that bystander behavior in relation to workplace bullying is related to organizational and social aspects of work. As an approach to reducing workplace bullying in the healthcare sector, focusing on bystander behavior has been identified as a promising intervention strategy (Illing et al., 2013). This is especially relevant in the healthcare context where employees are encouraged to speak up when critical incidents and errors are witnessed (Okuyama et al., 2014; Thompson et al., 2020). However, for bystander intervention to succeed, it is vital to establish a safe and supportive workplace and to ensure the engagement of management in the issue. Thus, organizations should have clear policies regarding how to report workplace bullying and the kinds of support available to both targets and bystanders. Overall, bystander behavior is not an individual “trait” that is independent of context. Rather, the work environment can either promote or discourage bystander intervention.

## AUTHOR CONTRIBUTIONS

*The conception and design of the study, acquisition of data, analysis, and interpretation of data*: Sandra Jönssonn and Tuija Muhonen. *Drafting the article or revising it critically for important intellectual content*: Sandra Jönssonn and Tuija Muhonen. *Final approval of the version to be submitted*: Sandra Jönssonn and Tuija Muhonen.

## CONFLICTS OF INTEREST

The authors declare no conflicts of interest.

## ETHICS STATEMENT

The study was approved by the Swedish Ethical Review Authority (2018‐00228).

## Data Availability

Due to the sensitive topic of the research, research data are not shared.
